# RCANs regulate the convergent roles of NFATc1 in bone homeostasis

**DOI:** 10.1038/srep38526

**Published:** 2016-12-05

**Authors:** Jung Ha Kim, Kabsun Kim, Inyoung Kim, Semun Seong, Byung-chul Jeong, Kwang-Il Nam, Kyung Keun Kim, Jeffery D. Molkentin, Nacksung Kim

**Affiliations:** 1 Department of Pharmacology, Chonnam National University Medical School, Gwangju 61469, Republic of Korea; 2 Department of Biomedical Sciences, Chonnam National University Medical School, Gwangju 61469, Republic of Korea; 3 Department of Anatomy, Chonnam National University Medical School, Gwangju 61469, Republic of Korea; 4 Cincinnati Children’s Hospital Medical Center, Department of Pediatrics, University of Cincinnati, Cincinnati 45247, OH, USA

## Abstract

Activation of calcineurin-dependent nuclear factor of activated T cells c1 (NFATc1) is convergent for normal bone homeostasis. NFATc1 regulates both osteoclastogenesis and osteoblastogenesis. Here we investigated the roles of regulator of calcineurin (RCAN) genes in bone homeostasis. RCANs function as potent physiological inhibitors of calcineurin. Overexpression of RCANs in osteoclast precursor cells attenuated osteoclast differentiation, while their overexpression in osteoblasts enhanced osteoblast differentiation and function. Intriguingly, opposing effects of RCANs in both cell types were shown by blocking activation of the calcineurin-NFATc1 pathway. Moreover, the disruption of RCAN1 or RCAN2 in mice resulted in reduced bone mass, which is associated with strongly increased osteoclast function and mildly reduced osteoblast function. Taken together, RCANs play critical roles in bone homeostasis by regulating both osteoclastogenesis and osteoblastogenesis, and they serve as inhibitors for calcineurin-NFATc1 signaling both *in vivo* and *in vitro*.

Bone is constantly renewed through the dynamic process of bone remodeling, maintaining healthy bone. This is delicately controlled by osteoclasts and osteoblasts, two functionally opposing cells^1^. Bone-resorbing osteoclasts are derived from monocyte/macrophage lineage cells dependent on two essential osteoclastogenic cytokines, macrophage colony-stimulating factor (M-CSF) and receptor activator of nuclear factor-κB (NF-κB) ligand (RANKL)^2^,^3^. Bone-forming osteoblasts differentiate from multipotent mesenchymal progenitors of bone marrow. In addition to bone formation, osteoblasts support osteoclast differentiation through production of M-CSF and RANKL^4^,^5^,^6^. The intricate balance between bone resorption and bone formation is vital for maintenance of normal bone homeostasis.

Nuclear factor of activated T cells c1 (NFATc1) may be profoundly involved in the regulation of bone homeostasis as it controls both osteoclast and osteoblast differentiation^7^,^8^,^9^,^10^,^11^,^12^,^13^. When NFATc1 is dephosphorylated by the cytoplasmic calcium/calmodulin/calcineurin complex, NFATc1 is activated and translocates to the nucleus where it regulates its target genes transcription in cooperation with other transcription factors^14^,^15^. Calcineurin-NFATc1 signaling pathways are indispensable for osteoclast differentiation and function^7^,^16^,^17^.

Osteoclast differentiation is reduced in osteoclast precursors treated with the calcineurin inhibitors cyclosporine A and FK506^7^. Young mice lacking NFATc1 develop osteopetrosis owing to defects in osteoclast differentiation^18^. Additionally, various studies have elucidated that NFATc1 upregulates expression of osteoclast marker genes including tartrate-resistant acid phosphatase (TRAP), osteoclast-associated receptor (OSCAR), calcitonin receptor, and cathepsin K to induce osteoclast differentiation and bone resorption^18^,^19^,^20^,^21^,^22^. However, the roles of calcineurin-NFATc1 signaling pathways in osteoblasts are still controversial. Several studies using mice with global disruption or activation of calcineurin-NFAT signaling suggested a positive role of calcineurin and NFAT in osteoblast differentiation and bone formation, while other studies using mice with conditional disruption of calcineurin B1 in osteoblasts or treated with cyclosporine A suggested calcineurin and NFAT negatively regulate osteoblast differentiation and bone formation^9^,^10^,^12^,^15^.

There are three regulators of calcineurin (RCAN) genes: RCAN1, RCAN2, and RCAN3, which inhibit calcineurin activity^23^,^24^,^25^,^26^. Of note, RCAN1 and RCAN2 directly bind to the calcineurin catalytic A subunit without preventing interaction with the regulatory B subunit or calmodulin and thereby inhibit the calcineurin phosphatase activity^25^. Although RCAN genes are potent endogenous inhibitors of calcineurin-NFAT signaling, several recent studies have shown that RCAN genes may facilitate calcineurin activity under certain some stress condition^27^. Therefore, the effects of RCAN genes on calcineurin-NFAT signaling may be dependent on cell type or microenvironment condition.

In this study, to further clarify the effect of RCAN genes on calcineurin-NFAT signaling and controversy over the role of NFATc1 in osteoblasts, we investigated how RCAN genes regulate differentiation and function of the two opposing bone cells *in vitro* and *in vivo*.

## Results

### RCANs negatively regulate RANKL-induced osteoclast differentiation

**Figure 1:**
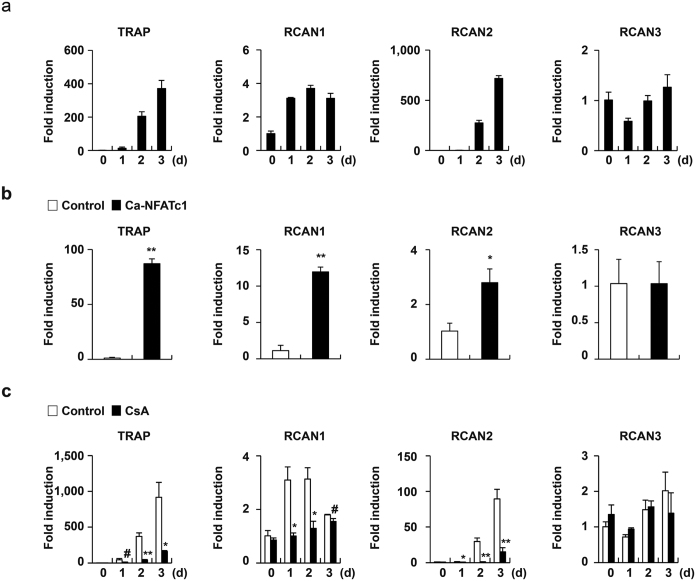
All RCAN genes are expressed during osteoclastogenesis. (**a**) BMMs were cultured in the presence of M-CSF and RANKL for the indicated times. (**b**) BMMs were transduced with pMX-IRES-EGFP (Control) or constitutively active NFATc1 (Ca-NFATc1) retrovirus and cultured in the presence of M-CSF for 4 days. (**c**) BMMs were cultured in the presence of M-CSF and RANKL with or without cyclosporine A (CsA, 5 μg/ml) for the indicated times. (**a**–**c**) Total RNA was harvested from cultured cells, and real-time PCR was performed to analyze expression of TRAP, RCAN1, RCAN2, and RCAN3. Data represent the mean ± SD of triplicate samples. #p < 0.05, *p < 0.01, **p < 0.001 *vs*. control, n = 3.

Because each RCAN is differentially expressed in various tissues, we firstly examined expression of RCANs during RANKL-induced osteoclast differentiation^28^,^29^,^30^. All RCANs including RCAN1, 2, and 3 were expressed in TRAP positive multinucleated osteoclasts [TRAP(+) MNCs] (Fig. 1a). Expression of RCAN1 and RCAN2, but not RCAN3 was increased as osteoclast precursor cells differentiate into mature osteoclasts (Fig. 1a). Next, we investigated whether NFATc1, a master transcription factor, regulates the expression of RCANs during osteoclastogenesis. Overexpression of constitutively active NFATc1 (Ca-NFATc1) in bone marrow-derived macrophage-like cells (BMMs) significantly increased expression of RCAN1 and RCAN2, but not of RCAN3 (Fig. 1b). We confirmed that expression of TRAP, known to be a down-stream target gene of NFATc1, was strongly increased by overexpression of Ca-NFATc1 (Fig. 1b). Additionally, treatment with the calcineurin inhibitor cyclosporine A during RANKL-induced osteoclast differentiation inhibited expression of RCAN1 and RCAN2 as well as TRAP (Fig. 1c). These results indicate that calcineurin-NFATc1 signaling cascade results in transcriptional activation of RCAN1 and RCAN2 but not RCAN3 during RANKL-mediated osteoclastogenesis.

**Figure 2:**
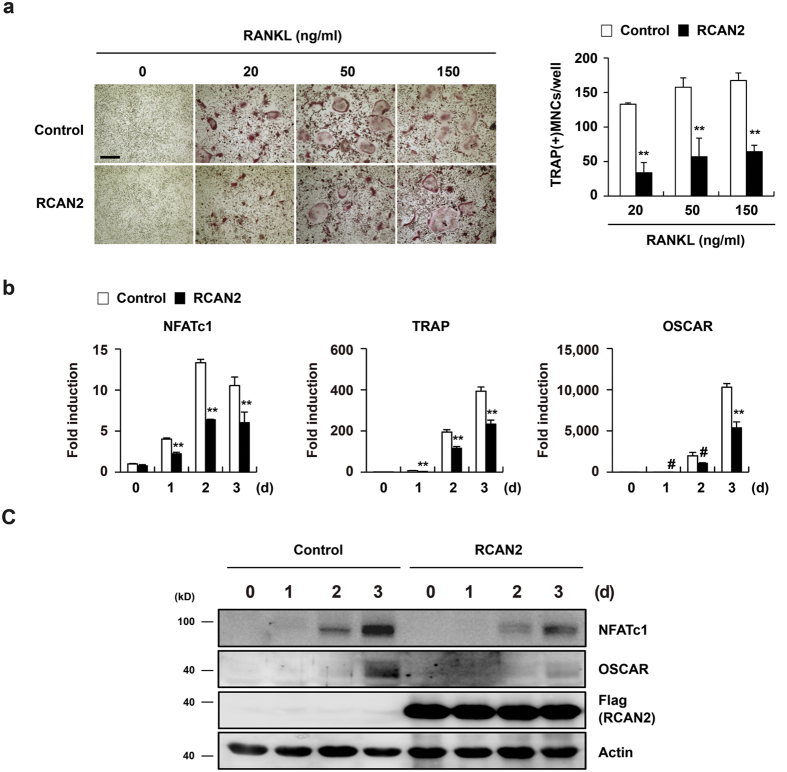
RCAN2 inhibits RANKL-induced osteoclast differentiation. (**a**–**c**) BMMs were transduced with pMX-IRES-EGFP (Control) or RCAN2 retrovirus and cultured in the presence of M-CSF and RANKL for 3 days. (**a**) Cultured cells were fixed and stained for TRAP (left panel). Numbers of TRAP(+) MNCs were counted (right panel). **p < 0.001 *vs*. control. Bar: 200 μm, n = 3. (**b**) Total RNA was collected at the indicated time points. Real-time PCR was performed to analyze expression of NFATc1, TRAP, and OSCAR. Data represent the mean ± SD of triplicate samples. #p < 0.05, **p < 0.001 *vs*. control, n = 3. (**c**) Cells were harvested at the indicated time points. Cell lysates were analyzed by Western blot analysis using antibodies specific for NFATc1, OSCAR, Flag, and Actin. All gels were run under the same experimental conditions and the representative images were cropped and displayed. Full-length blots are presented in Supplementary Figure 8.

To investigate the role of RCANs in RANKL-induced osteoclast differentiation, we overexpressed RCAN1, RCAN2, and RCAN3 respectively in BMMs using retrovirus. The formation of TRAP(+) MNCs induced by RANKL was attenuated by overexpression of RCANs including RCAN1, RCAN2, and RCAN3 as compared to control (Fig. 2a, Supplementary Figure 1). These data suggest that all RCAN genes play negative roles in RANKL-mediated osteoclast differentiation. Next, we examined the effect of RCANs on expression of osteoclast-related genes during RANKL-induced osteoclastogenesis. Since RCANs have overlapping functions in osteoclasts and expression of RCAN2 among the RCAN genes was the most firmly induced by RANKL stimulation (Fig. 1a), we focused on RCAN2. As shown in Fig. 2b and c, RCAN2 overexpression strongly inhibited RANKL-mediated NFATc1 induction (Fig. 2b,c). The expression of NFATc1 downstream target genes, TRAP and OSCAR, was also inhibited by RCAN2 overexpression. Collectively, these data show that RCAN2 acts as a negative regulator during RANKL-induced osteoclast differentiation by attenuating NFATc1 induction.

### RCAN2 inhibits transcriptional activity of NFATc1 in osteoclasts

**Figure 3:**
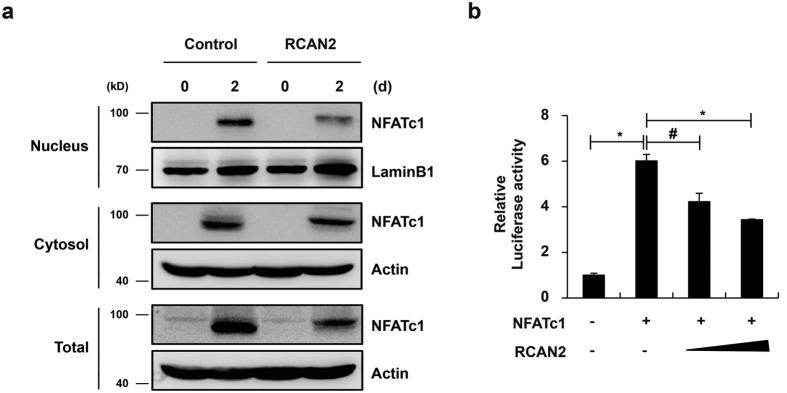
RCAN2 inhibits NFATc1 activation in osteoclasts. (**a**) BMMs were transduced with pMX-IRES-EGFP (Control) or RCAN2 retrovirus and cultured in the presence of M-CSF and RANKL for the indicated times. Western blot analysis was performed to analyze NFATc1 expression in nuclear, cytoplasmic, and whole cell fractions. All gels were run under the same experimental conditions and the representative images were cropped and displayed. Full-length blots are presented in Supplementary Figure 9. (**b**) An OSCAR luciferase reporter was co-transfected with NFATc1 and increasing amounts of RCAN2 into 293 T cells. Luciferase activity was measured a dual-luciferase reporter assay system. Data represent the mean ± SD of triplicate samples. #p < 0.05, *p < 0.01 *vs*. the control, n = 3.

Direct interaction of RCAN2 with calcineurin blocks NFAT-regulated gene expression^25^. Therefore, we investigated whether RCAN2 regulates calcineurin-mediated NFATc1 translocation into the nucleus during RANKL-induced osteoclastogenesis. Overexpression of RCAN2 attenuated RANKL-mediated NFATc1 induction in total cell extracts similar with results of Fig. 2c (Fig. 3a). The reduction in NFATc1 levels by RCAN2 overexpression was more clearly observed in nuclear than cytosolic fractions (Fig. 3a). Further, when a reporter plasmid containing a 1.7-kb OSCAR promoter region was cotransfected with NFATc1 into 293 T cells, relative luciferase activity was increased, while addition of RCAN2 strongly inhibited this activity (Fig. 3b). Therefore, these results indicate that RCAN2 can act as an inhibitor of RANKL-mediated calcineurin-NFATc1 signaling and thereby decreases expression of NFATc1-regulated genes including NFATc1 itself, TRAP, and OSCAR.

### RCANs enhance osteoblast differentiation and function

**Figure 4:**
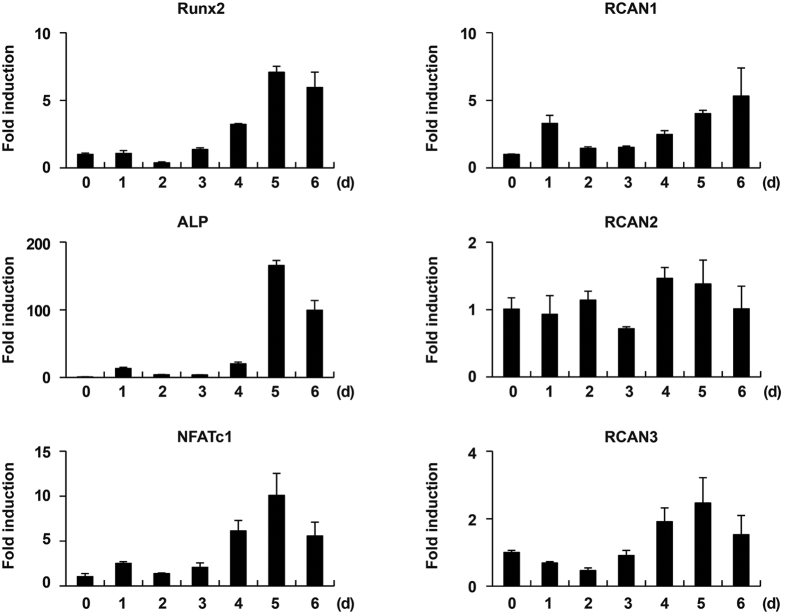
All RCAN genes expressed during osteoblastogenesis. Primary osteoblast precursor cells were cultured in osteogenic medium (OGM) containing BMP2, ascorbic acid, and β-glycerophosphate for the indicated times. Total RNA was collected at each time point, and real-time PCR was performed to assess the expression of Runx2, ALP, NFATc1, RCAN1, RCAN2, and RCAN3. Data represent the mean ± SD of triplicate samples, n = 3.

**Figure 5:**
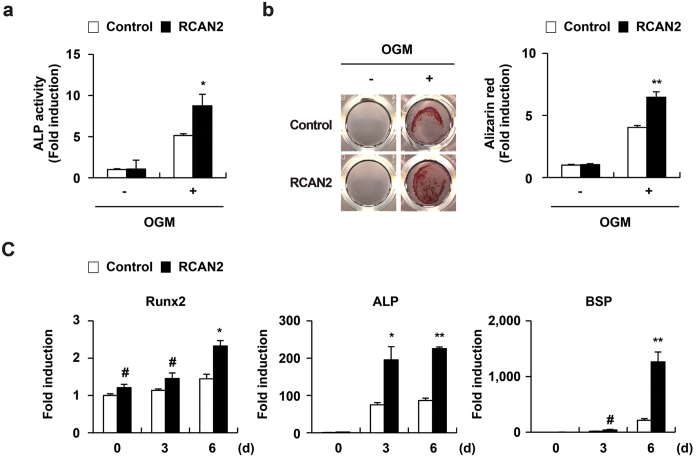
RCAN2 enhances osteoblast differentiation. (**a**–**c**) Osteoblasts were transduced with pMX-IRES-EGFP (control) or RCAN2 retrovirus and cultured in osteogenic medium (OGM). (**a**) Cells cultured for 3 days were subjected to the alkaline phosphatase activity (ALP) assay. *p < 0.01 *vs*. the control, n = 3. (**b**) Cells cultured for 9 days were fixed and stained for Alizarin red (left panel). Alizarin red staining activities was quantified by densitometry at 562 nm (right panel). **p < 0.001 *vs*. control, n = 3. (**c**) Cells were cultured for the indicated times, and real-time PCR was performed to analyze expression of Runx2, ALP, and BSP. Data represent the mean ± SD of triplicate samples. #p < 0.05, *p < 0.01, **p < 0.001 *vs*. the control, n = 3.

We next examined the roles of RCAN genes in osteoblasts, another bone cell lineage. When primary calvarial osteoblasts were cultured with osteogenic factors containing ascorbic acid, β-glycerophosphate, and BMP2, osteoblasts differentiated with increase in expression of osteoblast marker genes such as Runx2 and alkaline phosphatase (ALP) (Fig. 4). Expression of RCAN1, RCAN3, and NFATc1 was slightly increased as the cells differentiated, while RCAN2 expression remained constant during osteoblastogenesis (Fig. 4). Among the RCAN genes, only RCAN1 expression was regulated by the calcineurin-NFATc1 signaling cascade during osteoblastogenesis (Supplementary Figure 2). To evaluate osteoblast differentiation and function, we analyzed ALP activity and bone nodule formation respectively. Overexpression of all three RCANs in osteoblasts significantly increased ALP activity and bone nodule formation (Fig. 5a,b, Supplementary Figure 3a,b). The function of RCAN2 in osteoblasts was confirmed by upregulation of osteoblast marker genes such as Runx2, ALP, and bone sialoprotein (BSP) in RCAN2-infected osteoblasts (Fig. 5c). Interestingly, overexpression of Ca-NFATc1 in osteoblasts showed a contradictory effect to that of ectopic expression of RCANs (Supplementary Figure 4). Therefore, these results indicate that RCANs play important roles in osteoblast differentiation and function, via blocking negative effects of NFATc1 on osteoblast differentiation and function.

### RCAN2 is a negative regulator of NFATc1 in osteoblasts

**Figure 6:**
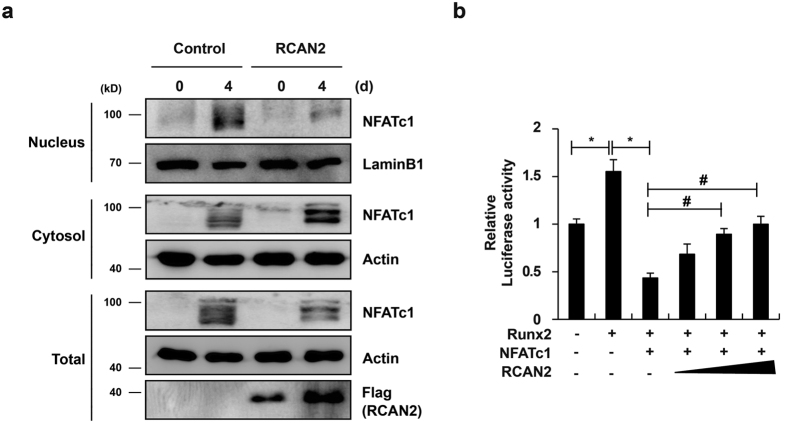
RCAN2 inhibits NFATc1 activation in osteoblasts. (**a**) Osteoblasts were transduced with pMX-IRES-EGFP (control) or RCAN2 retrovirus and cultured in osteogenic medium (OGM) for 4 days. Western blot analysis was performed to analyze NFATc1 expression in nuclear, cytoplasmic, and whole cell fractions. All gels were run under same experimental conditions and the representative images were cropped and displayed. Full-length blots are presented in Supplementary Figure 10. (**b**) An ALP luciferase reporter was co-transfected with the indicated plasmids expressing Runx2, NFATc1 and increasing amounts of RCAN2 into 293 T cells. Luciferase activity was measured using a dual-luciferase reporter assay system. Data represent the mean ± SD of triplicate samples. #p < 0.05, *p < 0.01 *vs*. control, n = 3.

Because both RCAN2 and NFATc1 are expressed in osteoblasts and overexpression of the two genes resulted in opposing effect on osteoblast differentiation and function, we tested whether RCAN2 regulates NFATc1 function in osteoblasts. When osteoblasts were cultured with osteogenic factors, both NFATc1 expression and nuclear translocation were strongly induced (Fig. 6a). However, RCAN2 overexpression not only inhibited NFATc1 expression but also its translocation into the nucleus. Furthermore, NFATc1 inhibited Runx2-induced ALP luciferase activity and these inhibitory effects were partially rescued by RCAN2 addition in a dose dependent manner (Fig. 6b). These data suggest that activated calcineurin-NFATc1 signaling negatively regulates osteoblast differentiation, perhaps through reducing Runx2 transcriptional activity and RCAN2 acts as an inhibitor of calcineurin-NFATc1 signaling in osteoblasts.

### RCAN2 knockout mice showed reduced bone mass

**Figure 7:**
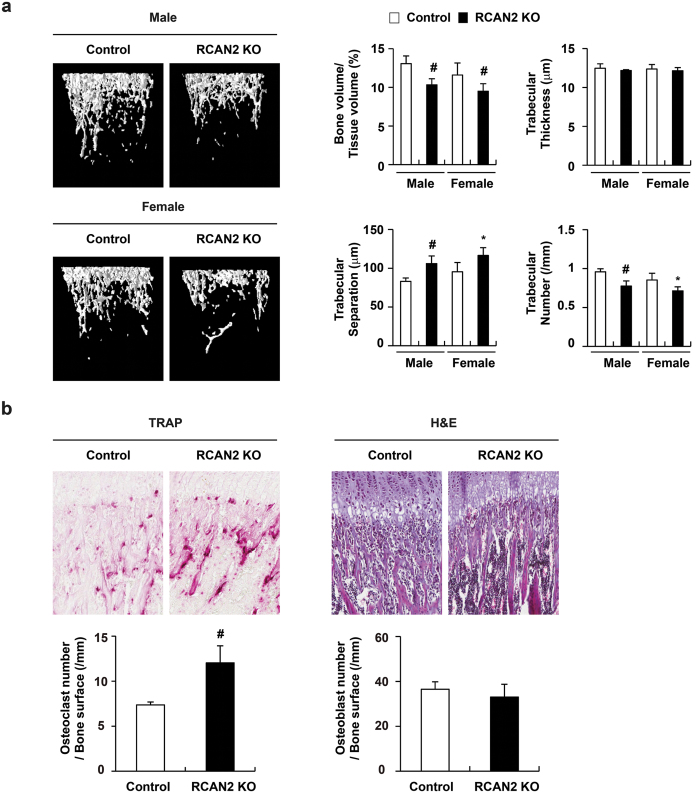
RCAN2 knockout mice exhibit reduced bone mass. (**a**) Representative three-dimensional images of femurs in control or RCAN2 knockout mice (left panel). Bone volume per tissue volume, trabecular bone thickness, trabecular separation, and trabecular number were assessed from the μCT measurements (right panel). #p < 0.05, *p < 0.01 *vs*. control, n = 3 (Male), n = 6 (Female). (**b**) Hematoxylin/eosin (H&E) and TRAP staining of histological sections of proximal tibiae (upper panel). Osteoclast numbers per bone surface and osteoblast numbers per bone surface were assessed (lower panel). #p < 0.05 *vs*. control, n = 3.

To investigate whether RCAN2 has an effect on bone homeostasis, we analyzed the bone phenotype of 4–5-week-old RCAN2 knockout mice using micro-computed tomography (μCT) imaging. As shown in Fig. 7a, RCAN2 knockout mice displayed reduced bone mass with decreased bone volume and trabecular numbers, as well as increased trabecular bone separation (Fig. 7a). Osteoclast number is significantly increased in RCAN2 knockout mice compared to control littermate mice (Fig. 7b) and the RANKL-induced osteoclast formation was significantly enhanced in BMM cells derived from RCAN2 knockout mice (Supplementary Figure 5). However, osteoblast number was slightly reduced in RCAN2 knockout mice without significance when compared to that of control littermate mice (Fig. 7b). Histomorphometric analysis revealed that the reduced bone mass in RCAN2 knockout mice resulted from increased osteoclast rather than decreased osteoblast function (Fig. 7b). Similar results were obtained from analysis of RCAN1 knockout mice (Supplementary Figure 6). Our observations collectively suggest that regulation of calcineurin-NFATc1 signaling by RCAN1 or RCAN2 may contribute to maintenance of normal bone homeostasis under physiological conditions.

## Discussion

Calcineurin-NFATc1 signaling is essential and sufficient for osteoclast differentiation, while the roles of calcineruin-NFATc1 signaling in osteoblasts are still controversial^7^,^8^,^9^,^10^,^11^,^12^,^15^,^18^. To verify the roles of calcineurin-NFATc1 signaling in osteoblasts, previous studies used calcineurin inhibitors *in vitro* or murine animal models, where calcineurin-NFATc1 signaling may be regulated by multiple factors^10^. To further clarify the direct effect of calcineurin-NFATc1 signaling on osteoblasts, we overexpressed Ca-NFATc1 in osteoblasts. Compared to the positive role of NFATc1 in osteoclasts, overexpression of Ca-NFATc1 in osteoblasts significantly inhibited osteoblast differentiation as well as bone nodule formation (Supplementary Figure 4). Further, we discovered that although future study will be required to elucidate the precise molecular mechanism, NFATc1 blocked not only Runx2 transcriptional activity but also expression of Runx2 target genes, including Runx2 itself, ALP, and BSP (Supplementary Figure 4). Therefore, our findings indicate that ectopic expression of NFATc1, when limited to osteoblasts, has a negative effect on osteoblast differentiation and function.

Here we present multiple lines of evidence suggesting all RCAN genes have overlapping functions in osteoclasts and osteoblasts. The functions of RCANs are to hinder osteoclast differentiation and facilitate osteoblast differentiation. These functions of RCANs oppose the activity of NFATc1 in both osteoclasts and osteoblasts. In addition, we observed that RCAN2 prevents association between calcineurin and NFATc1, resulting in reduced nuclear localization of NFATc1 (Figs 3 and 6, and Supplementary Figure 7). It is well known that RCAN1 and RCAN2 can bind to calcineurin, thereby inhibiting calcineurin-NFAT signaling^25^,^31^,^32^,^33^. Additionally, recent evidence indicates that RCAN3 also binds to calcineurin and blocks NFAT-dependent gene expression^34^. These findings, together with our present results, collectively suggest that all RCAN genes play important roles in the two bone cells through inhibition of calcineurin-NFATc1 signaling.

RANKL participates in negative and positive feedback loops to regulate osteoclast formation. For instance, we demonstrated a negative feedback loop involving NFATc1 during osteoclast differentiation in a previous study^35^. RANKL induces the expression of the MHC class II transactivator through NFATc1 induction and in turn, MHC class II transactivator inhibits osteoclast differentiation via downregulation of NFATc1 and OSCAR^35^. There are several instances during normal muscle development RCAN1 among RCAN genes act as an endogenous negative feedback regulation of calcineurin-NFAT signaling^24^. Since RANKL strongly induced expression of RCAN1 and RCAN2 but not RCAN3, we hypothesized that both RCAN2 and RCAN1 are negative feedback regulators during osteoclastogenesis (Fig. 1a). RANKL-mediated expression of RCAN1 and RCAN2 depends on activation of calcineruin-NFATc1 signaling, and RCAN2 negatively regulated RANKL-induced osteoclast differentiation via downregulation NFATc1. Therefore, this negative feedback regulation of the RANKL-NFATc1-RCAN axis contributes to regulation of osteoclast formation.

In a previous report, Bassett *et al*. reveled that juvenile RCAN2 knockout mice exhibited reduced bone mineral content in both humerus and vertebrae^36^. Although they did not precisely analyze the basic cause of reduced bone mineral content, their results may be consistent with our results observed from femoral bone analyses that RCAN2 deficiency causes dysregulation of osteoclast and osteoblast differentiation. However, they also reported that adult RCAN2 knockout mice exhibited increased bone mineralization due to normal bone resorption but reduced bone formation. The age-dependent alteration in the bone phenotype of RCAN2 knockout mice may be accompanied by a changed the effect of RCAN2 deficiency on osteoclasts. Indeed, although multiple studies verified a negative role of RCANs in calcineurin-NFATc1 signaling *in vivo*, various contradictory roles of RCANs have been also reported^14^. For example, RCAN1 knockout mice showed an impaired cardiac hypertrophic response to pressure overload accompanied by calcineurin activation^37^. RCANs may function differently depending on the target cell types or levels of calcineurin in certain microenvironments. In particular, the bone microenvironment can be modulated by various factors including aging, obesity, and inflammation, so RCANs effects on calcineurin-NFATc1 signaling may be dependent on these changes. In this study, we analyzed only juvenile RCAN1 or RCAN2 knockout mice under physiological condition. As bone homeostasis is very intricately controlled by various factors, additional study will be required to elucidate the effect of RCANs deficiency on bone homeostasis during age-related pathological conditions.

In summary, *in vitro*, RCANs negatively regulate calcineurin-NFATc1 signaling in osteoclasts and osteoblasts. In addition, RCANs are likely to function as inhibitors of calcineurin-NFATc1 *in vivo*, at least, under physiological bone condition. Therefore, RCANs play critical roles in bone homeostasis through regulation of calcineurin-NFATc1 signaling.

## Methods

### Mice

The RCAN1 and RCAN2 knockout mice have been described previously^27^. Heterozygous mice were crossed to generate knockout and wild-type offspring. All animal experiments were approved by the Chonnam National University Medical School Research Institutional Animal Care and Use Committee and were carried out in accordance with the approved guidelines.

### Retroviral gene transduction

The retroviral packaging cell line, PlatE, was maintained in Dulbecco’s modified Eagle’s medium (DMEM; HyClone Laboratories, Logan, UT) supplemented with 10% FBS, penicillin (100 U/mL), and streptomycin (100 mg/mL) in the presence of puromycin (1 μg/mL) and blasticidin (10 μg/mL). To prepare retroviral supernatants, retroviral vectors were transfected into Plat E cells using FuGENE 6 (Promega, Madison, WI) according to the manufacturer’s instructions. Viral supernatants were collected from the culture medium 48 hours after transfection. For retroviral infection, bone marrow-derived macrophages (BMMs) or osteoblast precursor cells were incubated with the viral supernatants for 6 hours in the presence of 10 μg/mL polybrene (Sigma-Aldrich).

### Osteoclast differentiation

Mouse bone marrow cells isolated from tibiae and femurs of 6-week-old mice were cultured in α-MEM (HyClone Laboratories) containing 10% FBS, 100 U/mL penicillin, and 100 mg/mL streptomycin (Life Technologies, Carlsbad, CA) in the presence of M-CSF (30 ng/ml) for 3 days. The attached BMMs were further cultured in the presence of M-CSF (30 ng/ml) and RANKL (20 ng/mL–150 ng/ml). Cultured cells were fixed with 3.7% formalin and stained for TRAP. TRAP-positive multinucleated cells (>3 nuclei/cell) were counted as osteoclasts.

### Osteoblast differentiation

Primary osteoblast precursor cells were isolated from newborn mouse calvaria by enzymatic digestion with 0.1% collagenase (Life Technologies) and 0.2% dispase II (Roche Diagnostics GmbH, Mannheim, Germany). For osteoblast differentiation, primary osteoblast precursor cells were cultured in osteogenic medium containing BMP2 (100 ng/ml), ascorbic acid (50 μg/ml), and β-glycerophosphate (100 mM). To assess osteoblast differentiation, osteoblast precursor cells cultured for 3 days were subjected to an alkaline phosphatase activity (ALP) assay. Cells were lysed using osteoblast lysis buffer [50 mM Tris-HCl (pH 7.4), 1% Triton X-100, 150 mM NaCl, and 1 mM EDTA]. The cell lysates were incubated with p-nitrophenyl phosphate substrate (Sigma-Aldrich), and ALP activity was measured with a spectrophotometer in absorbance at 405 nm. For the mineralization assay, cells were induced for 9–12 days and then fixed with 70% ethanol and stained with 40 mM Alizarin red (pH 4.2). After the nonspecific staining was removed with PBS, Alizarin red staining was visualized with a CanoScan 4400 F (Canon Inc., Japan). To quantify matrix calcification, Alizarin red stained cells were incubated with 10% cetylpyridinium chloride solution for 30 minutes at room temperature, and solution was measured in absorbance at 562 nm.

### Real-time PCR

Total RNA was isolated from cultured cells using Qiazol reagent (Qiagen), and 2 μg of the isolated RNA was reverse transcribed into cDNA using Superscript II Reverse Transcriptase (Invitrogen). Quantitative real-time PCR analysis was performed in triplicate with a Rotor-Gene Q (Qiagen) with SYBR Green (Qiagen). Quantification was normalized to the amounts of endogenous GAPDH. The primers used for real-time PCR were: GAPDH, 5′-TGA CCA CAG TCC ATG CCA TCA CTG-3′ and 5′-CAG GAG ACA ACC TGG TCC TCA GTG-3′; RCAN1, 5′-AGG CTG CGG CTG CAC AAG ACC GAG-3′ and 5′-CAC TGG GAG TGG TGT CTG TCG CTG-3′; RCAN2, 5′-TAT GAT GAA TGT GTG ACG TTC CAG-3′ and 5′-TGT AGA CTC AGT TCC AGC GTG CAG-3′; RCAN3, 5′-GAG TTC CAC GGA CGG AAG CTG AAG-3′ and 5′-TTC AGT TTC GCT CTC ACA GAC GTG-3′; NFATc1, 5′-CTC GAA AGA CAG CAC TGG AGC AT-3′ and 5′-CGG CTG CCT TCC GTC TCA TAG-3′; TRAP, 5′-CTG GAG TGC ACG ATG CCA GCG ACA-3′ and 5′-TCC GTG CTC GGC GAT GGA CCA GA-3′; OSCAR, 5′-TGC TGG TAA CGG ATC AGC TCC CCA GA-3′ and 5′-CCA AGG AGC CAG AAC CTT CGA AAC T-3′; Runx2, 5′-CCC AGC CAC CTT TAC CTA CA-3′ and 5′-CAG CGT CAA CAC CAT CAT TC-3′; Alp, 5′-CAA GGA TAT CGA CGT GAT CAT G-3′ and 5′-GTC AGT CAG GTT GTT CCG ATT C-3′; BSP, 5′-GGA AGA GGA GAC TTC AAA CGA AG-3′ and 5′-CAT CCA CTT CTG CTT CTT CGT TC-3′.

### Micro-computed tomography (μCT) analysis

μCT analysis was performed as previously described^38^. The collected distal femurs were fixed overnight in 75% ethanol. μCT images were scanned by using a high-resolution Skyscan 1172 system (Skyscan, Kontich, Belgium) at 50 kV and 201 μA with a 0.5 mm aluminum filter and a resolution of 11 μm pixel^−1^. Images were captured every 0.7° over an angular range of 180°. Raw images were reconstructed into serial cross sections and femoral morphometric parameters were analyzed by using image reconstruction software (NRecon 1.4, Skyscan), data analysis software (CTAn, Skyscan), and three-dimensional model visualization software (Ant 2.4, Skyscan).

### Western blotting

Cultured cells were lysed in lysis buffer containing 50 mM Tris-HCl (pH 8.0), 150 mM NaCl, 1 mM EDTA, 0.5% Nonidet P-40, 1 mM PMSF, and protease inhibitor cocktails. Equal amounts of protein concentration of cell lysates were separated using SDS-PAGE and transferred to PVDF membrane (Millipore Corporation, Billerica, MA). Membranes were incubated with specific primary antibodies, and probed with a horseradish peroxidase conjugated secondary antibody. Signals were detected with ECL solution (Millipore Corporation) and analyzed by LAS3000 luminescent image analyzer (GE Healthcare, Piscataway, NJ).

### Bone histomorphometric analysis

Bone histomorphometric analysis was carried out following protocol that has been previously described^38^. The collected tibiae were fixed in 4% paraformaldehyde and decalcified in 5.5% EDTA buffer for 2 weeks at 4 °C. Samples were gradually dehydrated, embedded in paraffin, and cut into 4-μm-thick longitudinal sections. After the sections were deparaffinized using xylene, they were stained with hematoxylin and eosin (H&E) or TRAP to quantify osteoblasts and osteoclasts, respectively.

### Luciferase assay

293 T cells were plated in 24-well plates at a density of 2 × 10^4^ cells/well 24 hours prior to transfection. The reporter plasmid and various expression vectors were cotransfected into 293 T cells using FuGENE 6, according to the manufacturer’s protocol. After 48 hours transfection, cells were lysed in passive lysis buffer (Promega). Luciferase activity was measured using a dual-luciferase reporter assay system (Promega) according to the manufacturer’s instructions.

### Statistics

All values are expressed as the mean ± SD. Statistically significant differences were determined using a two-tailed Student’s *t-*test for two independent samples. Differences exhibiting p < 0.05 were considered statistically significant.

## Additional Information

**How to cite this article**: Kim, J. H. *et al*. RCANs regulate the convergent roles of NFATc1 in bone homeostasis. *Sci. Rep.* **6**, 38526; doi: 10.1038/srep38526 (2016).

**Publisher's note:** Springer Nature remains neutral with regard to jurisdictional claims in published maps and institutional affiliations.

## Supplementary Material

Supplementary Information
