# Supplementary material for: RCANs regulate the convergent roles of NFATc1 in bone homeostasis
Source: Sci Rep. 2016 Dec 5;6:38526. doi: 10.1038/srep38526 (PMC5137032; doi:10.1038/srep38526)
Supplement: Supplementary Information [file srep38526-s1.pdf]

RCANs regulate the convergent roles of NFATc1 in bone homeostasis

Jung Ha Kim[1,2], Kabsun Kim[1], Inyoung Kim[1], Semun Seong[1,2], Byung-chul Jeong[1,2], Kwang-Il Nam[3], Kyung Keun Kim[1], Jeffery D. Molkentin[4], Nacksung Kim[1,2,*]

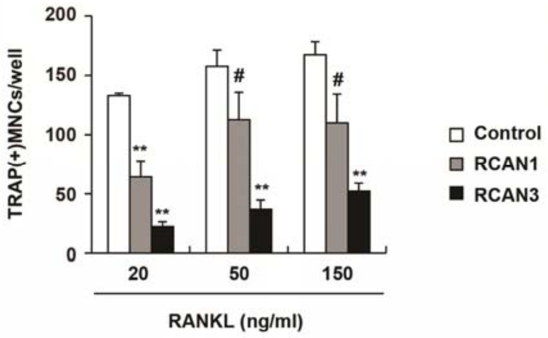

200
150
100
50
0
TRAP(+)MNCs/well
**
**
#
**
#
**
20
50
150
RANKL (ng/ml)
Control
RCAN1
RCAN3

Supplementary Figure 1. RCAN1 and RCAN3 attenuate RANKL-induced osteoclast differentiation. BMMs were transduced with pMX-IRES-EGFP (Control), RCAN1, or RCAN3 retrovirus and cultured in the presence of M-CSF and RANKL for 3 days. Cultured cells were fixed and stained for TRAP and numbers of TRAP(+) MNCs were counted. #$p < 0.05$, **$p < 0.001$ *vs*. control, $n = 3$.

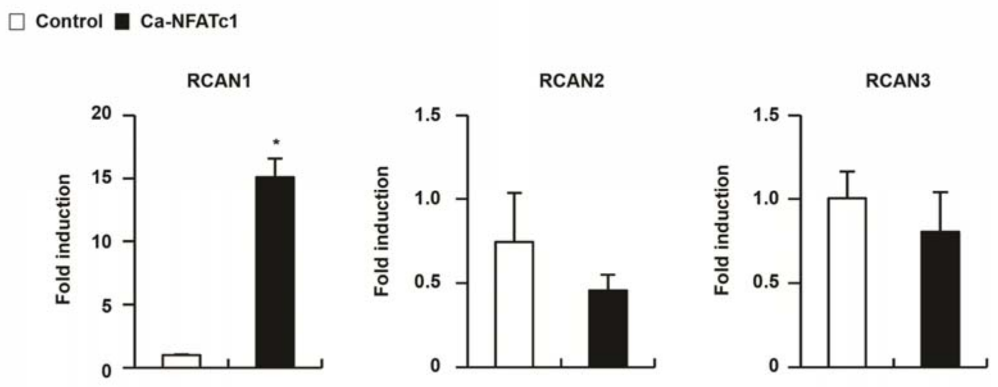

Control
Ca-NFATc1
RCAN1
RCAN2
RCAN3
Fold induction
20
15
10
5
0
*
1.5
1.0
0.5
0

Supplementary Figure 2. The calcineurin-NFATc1 signaling cascade regulates RCAN1 expression during osteoblast differentiation. MC3T3E1 cells were transduced with pMX-IRES-EGFP (Control) or constitutively active NFATc1 (Ca-NFATc1) retrovirus and cultured in osteogenic medium (OGM) containing BMP2, ascorbic acid, and β-glycerophosphate for 4 days. Total RNA was harvested from cultured cells, and real-time PCR was performed to analyze the expression of RCAN1, RCAN2, and RCAN3. Data represent the mean ± SD of triplicate samples. **$p < 0.001$ *vs*. control.

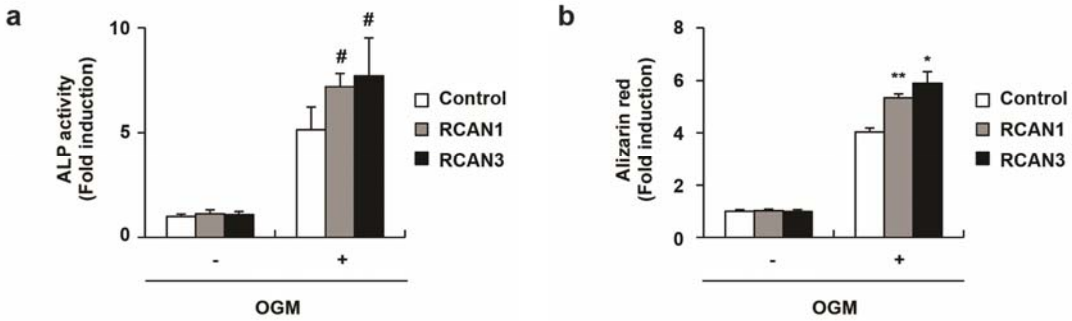

a
ALP activity
(Fold induction)
10
5
0
#
#
Control
RCAN1
RCAN3
-
+
OGM
b
Alizarin red
(Fold induction)
8
6
4
2
0
**
*
Control
RCAN1
RCAN3
-
+
OGM

Supplementary Figure 3. RCAN1 and RCAN3 enhance osteoblast differentiation. (a - b) Osteoblasts were transduced with pMX-IRES-EGFP (control), RCAN1, or RCAN3 retrovirus and cultured in osteogenic medium (OGM). (a) Cultured cells for 3 days were subjected to the alkaline phosphatase activity (ALP) assay. #p < 0.05 *vs*. control, n = 3. (b) Cultured cells for 9 days were fixed and stained for Alizarin red. Alizarin red staining activities was quantified by densitometry at 562 nm. *p < 0.01, **p < 0.001 *vs*. control, n = 3.

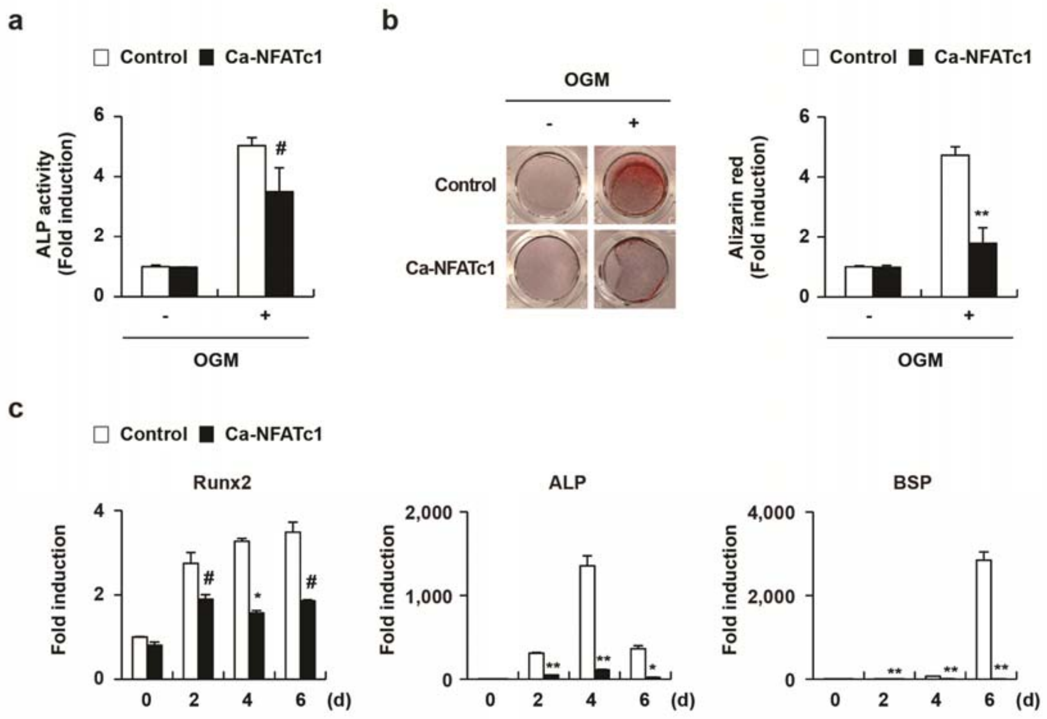

a
Control
Ca-NFATc1
ALP activity (Fold induction)
6
4
2
0
#
-
+
OGM
b
OGM
-
+
Control
Ca-NFATc1
Control
Ca-NFATc1
Alizarin red (Fold induction)
6
4
2
0
**
-
+
OGM
c
Control
Ca-NFATc1
Runx2
Fold induction
4
2
0
#
*
#
0
2
4
6
(d)
ALP
Fold induction
2,000
1,000
0
**
**
*
0
2
4
6
(d)
BSP
Fold induction
4,000
2,000
0
**
**
**
0
2
4
6
(d)

Supplementary Figure 4. Overexpression of Ca-NFATc1 inhibits osteoblast differentiation. (a – c) Osteoblasts were transduced with pMX-IRES-EGFP (control) or Ca-NFATc1 retrovirus and cultured in osteogenic medium (OGM). (a) Cells cultured for 3 days were subjected to the alkaline phosphatase activity (ALP) assay. #$p < 0.05$ *vs*. control, $n = 3$. (b) Cells cultured for 9 days were fixed and stained for Alizarin red (left panel). Alizarin red staining activities was quantified by densitometry at 562 nm (right panel). **$p < 0.001$ *vs*. control, $n = 3$. (c) Transduced cells were cultured for the indicated times, and Real-time PCR was performed for the expression of Runx2, ALP, and BSP. Data represent the mean ± SD of triplicate samples. #$p < 0.05$, *$p < 0.01$, **$p < 0.001$ *vs*. control, $n = 3$.

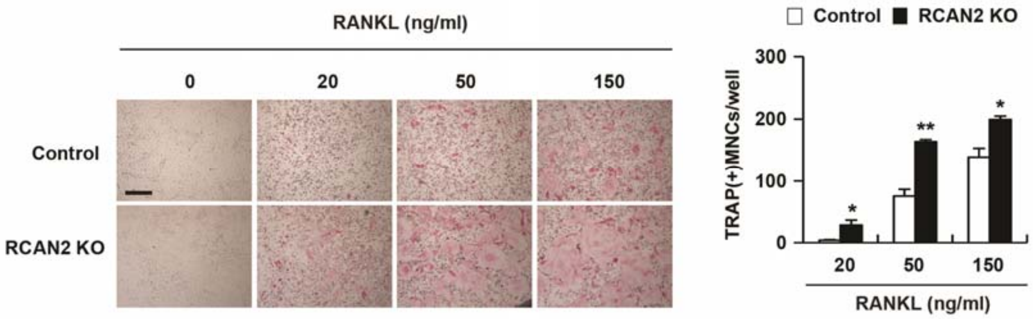
RANKL (ng/ml)
0
20
50
150
Control
RCAN2 KO
Control
RCAN2 KO
TRAP(+)MNCs/well
300
200
100
0
*
**
*
20
50
150
RANKL (ng/ml)

Supplementary Figure 5. RCAN2 deficiency enhances RANKL-induced osteoclast differentiation. Bone marrow cells harvested from the long bones of RCAN2 knockout mice (RCAN2 KO) and their wild-type littermates (Control). BMMs were cultured for 3 days with M-CSF and various concentrations of RANKL, as indicated. Cultured cells were stained to detect TRAP (left panel). The number of TRAP-positive multinucleated cells (MNCs) per well was counted (right panel). $*p < 0.01$ and $**p < 0.001$ vs. the control. Bar: 200 μm, $n = 3$.

a

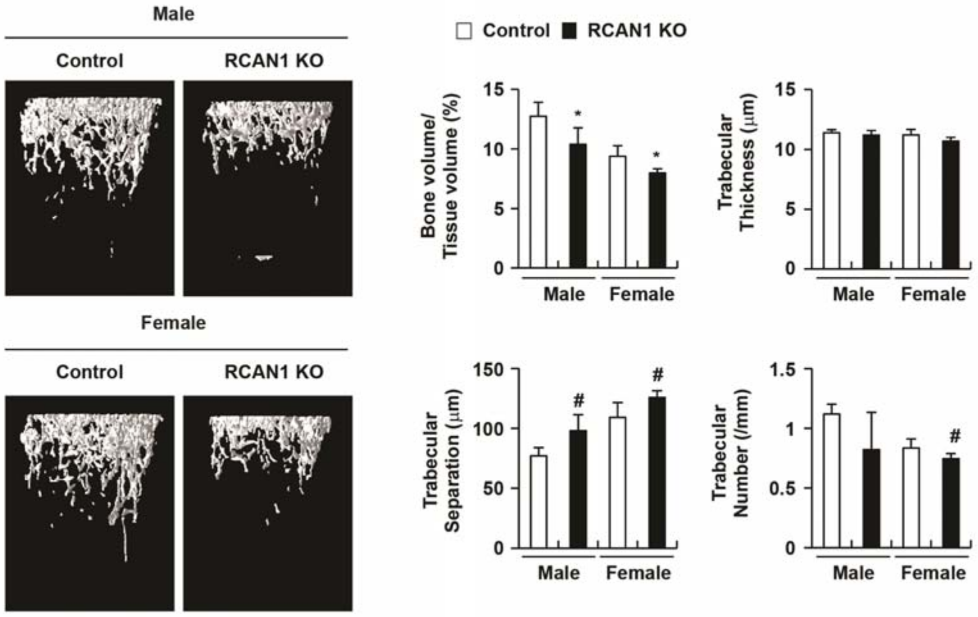
Male
Control
RCAN1 KO
Female
Control
RCAN1 KO
Control
RCAN1 KO
Bone volume/ Tissue volume (%)
Trabecular Thickness (μm)
Trabecular Separation (μm)
Trabecular Number (/mm)
Male
Female

b

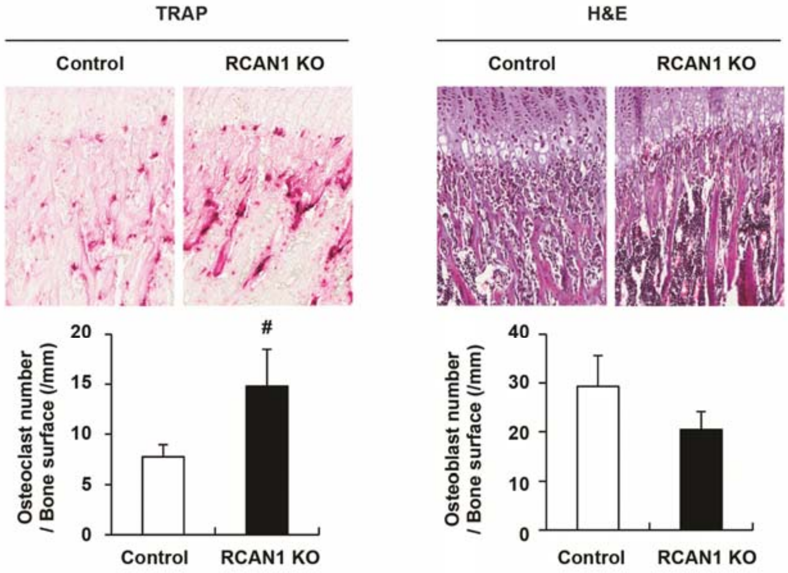
TRAP
H&E
Control
RCAN1 KO
Osteoclast number / Bone surface (/mm)
Osteoblast number / Bone surface (/mm)
Control
RCAN1 KO

Supplementary Figure 6. RCAN1 knockout mice exhibit reduced bone mass. (a) Representative three dimensional images of femurs in control or RCAN1 knockout mice (left panel). Bone volume per tissue volume, trabecular bone thickness, trabecular separation, and trabecular number were assessed from the μCT measurements (right panel). #$p < 0.05$, *$p < 0.01$ *vs*. control, n = 3 or 6 (Male), n = 5 or 7 (Female). (b) Hematoxylin/eosin (H&E) and TRAP staining of histological sections of proximal tibiae (upper panel). Osteoclast numbers per bone surface and osteoblast numbers per bone surface were assessed (lower panel). #$p < 0.05$ *vs*. control, n = 3 or 5.

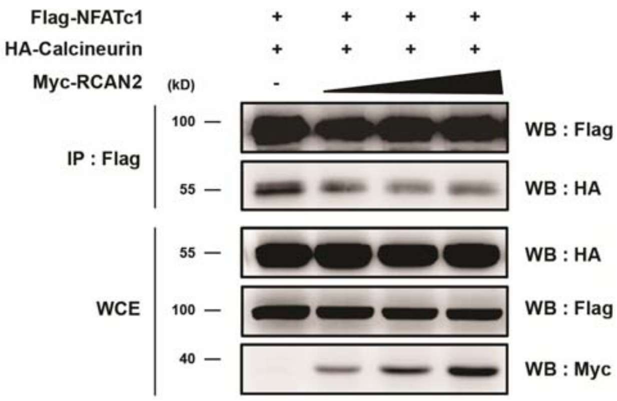
Flag-NFATc1
HA-Calcineurin
Myc-RCAN2
(kD)
IP : Flag
WCE
100
55
55
100
40
WB : Flag
WB : HA
WB : HA
WB : Flag
WB : Myc

Supplementary Figure 7. RCAN2 prevents interaction between calcineurin and NFATc1. 293T cells were co-transfected with plasmids expressing NFATc1, calcineurin, and RCAN2 as indicated. After 2 days of transfection, cell lysates were immunoprecipitated (IP) with Flag antibodies. Immnoprecipitated samples or whole cell extracts (WCE) were subjected to SDS-PAGE and Western blotting (WB) using anti-HA, anti-Flag, or anti-Myc antibodies. All gels were run under the same experimental conditions and representative images were cropped and displayed.

C

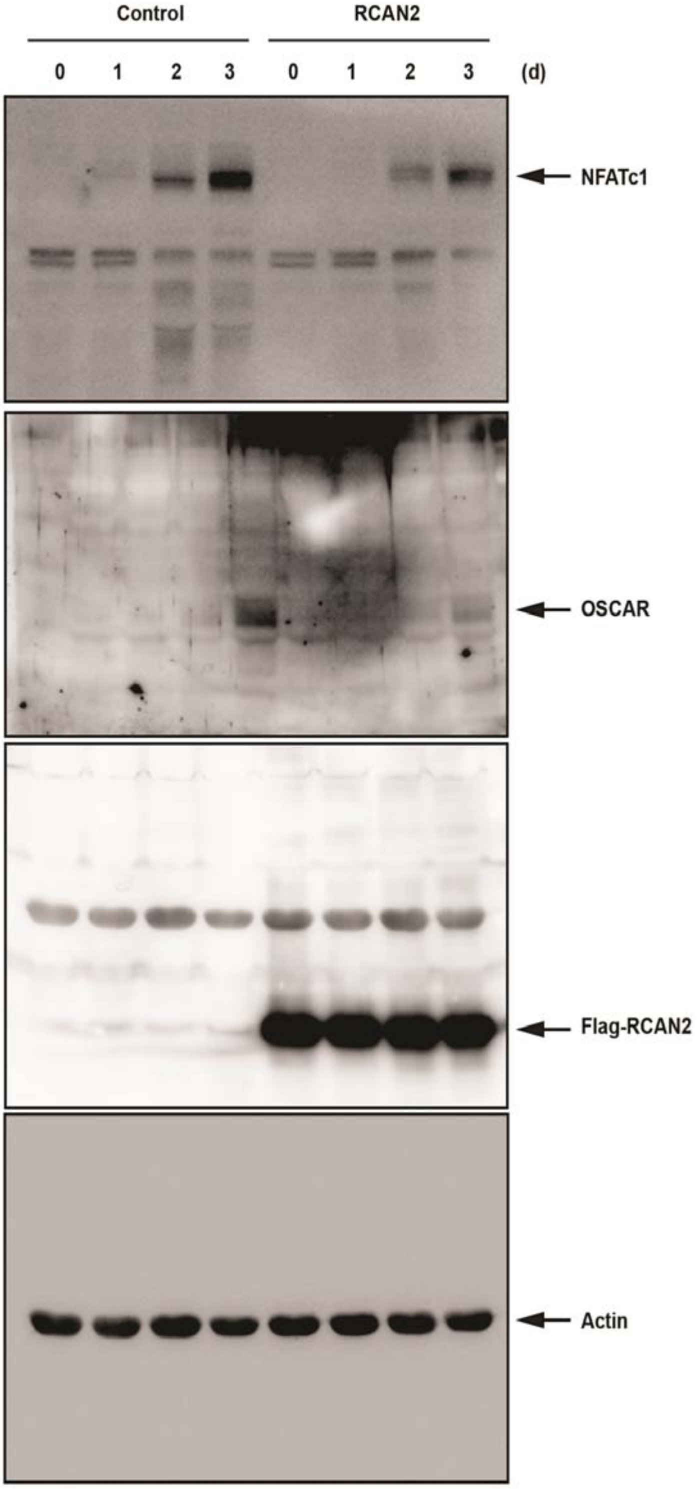

Supplementary Figure 8. Full-length images of immunoblots shown in Figure 2c.

a

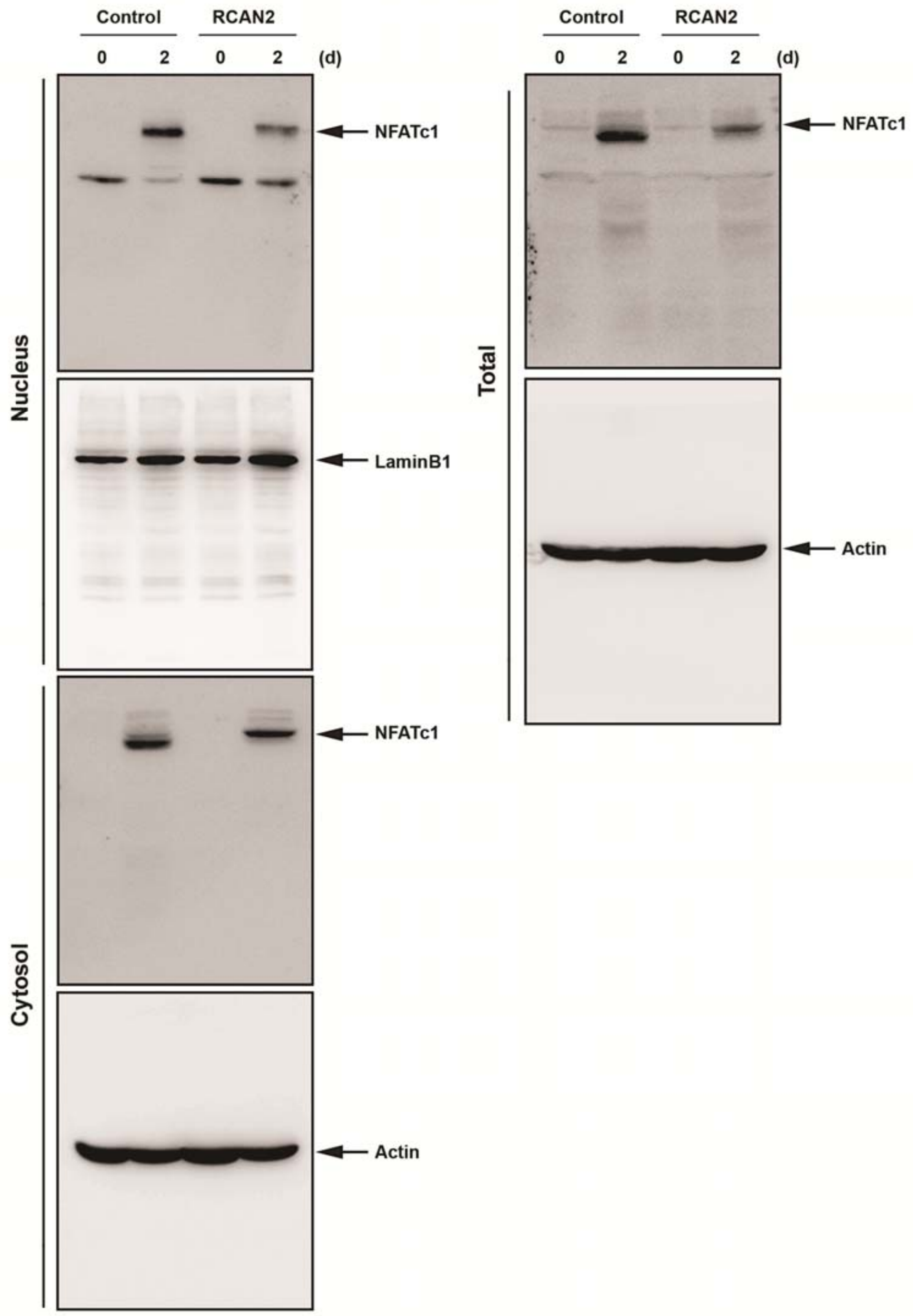
Control
RCAN2
0
2
0
2
(d)
NFATc1
LaminB1
NFATc1
Actin
Nucleus
Cytosol
Control
RCAN2
0
2
0
2
(d)
NFATc1
Actin
Total

Supplementary Figure 9. Full-length images of immunoblots shown in Figure 3a.

a

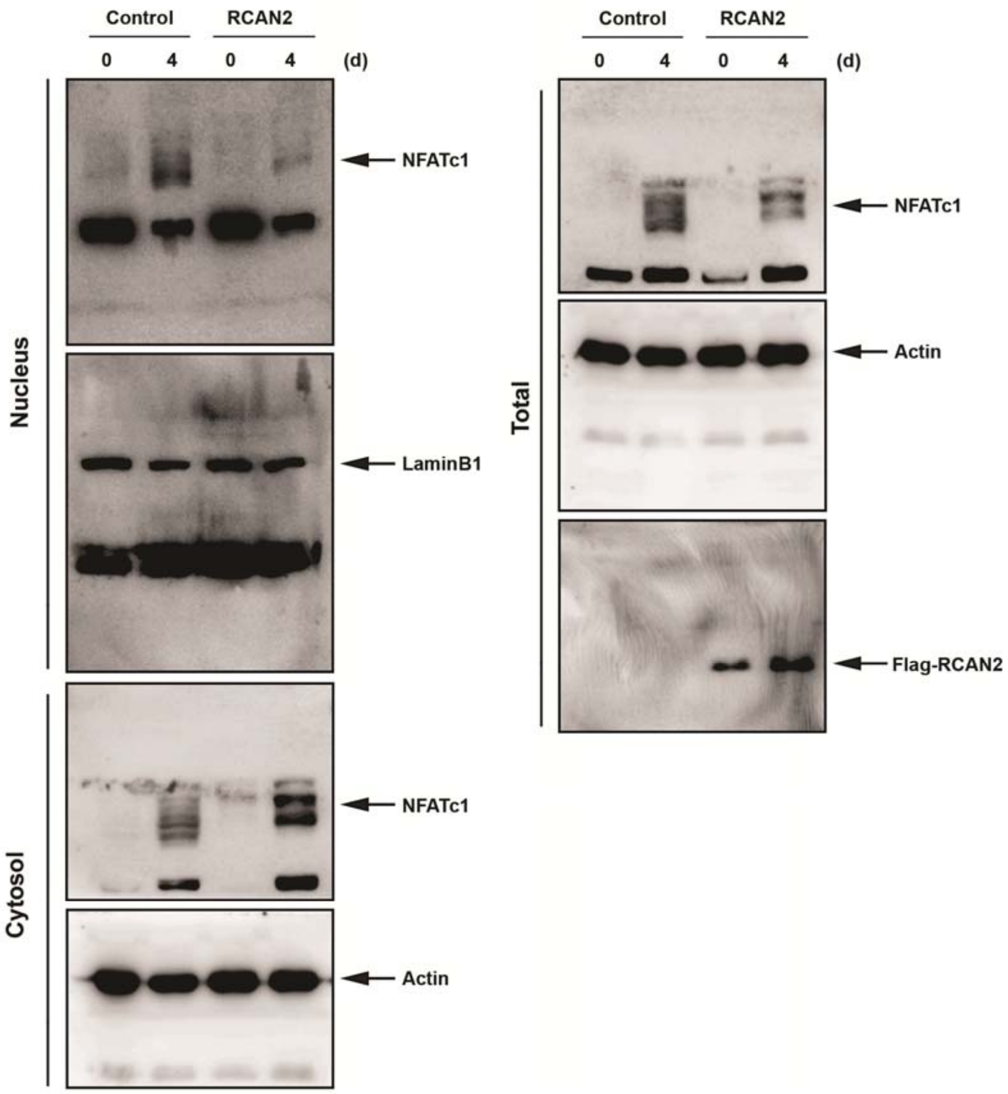

Control
RCAN2
0
4
0
4
(d)
Nucleus
NFATc1
LaminB1
Cytosol
NFATc1
Actin
Total
NFATc1
Actin
Flag-RCAN2

Supplementary Figure 10. Full-length images of immunoblots shown in Figure 6a.
